# TMA for all: a new method for the construction of tissue microarrays without recipient paraffin block using custom-built needles

**DOI:** 10.1186/1746-1596-1-14

**Published:** 2006-07-25

**Authors:** Andréa Rodrigues Cordovil Pires, Felipe da Matta Andreiuolo, Simone Rabello de Souza

**Affiliations:** 1 Fonte Medicina Diagnóstica Ltda. and Fluminense Federal University, Rio de Janeiro, Niterói, Brazil; 2 Fonte Medicina Diagnóstica Ltda., Rio de Janeiro, Niterói, Brazil

## Abstract

**Background:**

TMAs are becoming a useful tool for research and quality control methods, mostly for immunohistochemistry and *in situ *hybridization.

**Methods:**

A new technique that allows building TMA blocks with more than 300 tissue cores without using a recipient paraffin block for the tissue cores and without using a commercial TMA builder instrument is described. This technique is based on the construction of TMA needles modifying conventional hypodermic needles to punch tissue cores from donor blocks, which are attached by double-side adhesive tape on a computer-generated paper grid used to align the cores on the block mould, which is filled with liquid paraffin.

**Results:**

More than two hundred TMA blocks were constructed using this method, utilized in immunohistochemistry and histochemistry as positive and negative controls and also in research.

**Conclusion:**

This technique has the following advantages: it is easy to reproduce, affordable, quick and creates uniform blocks with more than 300 cores aligned, adherent and easy to cut, with negligible losses during cutting and immunohistochemistry and in situ hybridization procedures.

## Background

Tissue microarrays (TMA) are becoming a useful tool for research and quality control on immunohistochemistry (IHC) and in situ hybridization methods [1-3]. The conventional construction of a TMA block involves the use of a commercial TMA builder instrument to punch the cores from donor blocks, and the transference of these tissue cores to a recipient block, producing blocks with even 1000 tissue cores. This technique has some disadvantages:

1. as tissue cores from different donor blocks are placed inside holes in the recipient block, to create and improve the adherence between tissue cores and the recipient block, the TMA block has to be lightly heated to melt both. During this phase cores may misalign or the fusion may not be complete, resulting in core losses;

2. the need for adhesive tape during microtome cutting by some commercial available TMA devices;

3. the cores may be inserted too down in the recipient block, resulting in absence of the most profound cores in the first cuts and,

4. the commercial TMA builder instrument and its disposable needles are not affordable to many Pathology laboratories worldwide.

This study was carried out in order to produce TMA blocks without the disadvantages listed above.

## Methods

### Needles (figure 1)

**Figure 1:**
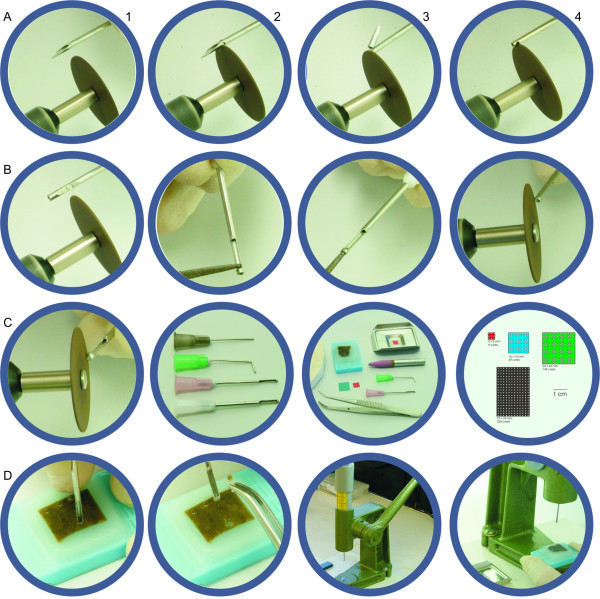
**Needle customization and paraffin block punching**. Needle customization, step by step – cutting the tip (A1 to A3), opening the lateral window (A4 to B2), creating a new bevel (B3 to C1) and custom-built needles (C2); TMA production kit (C3); paper grids (C4); paraffin block punching, manual (D1 and D2) and with hand-press grommet inserting machine (D3 and D4).

Conventional hypodermic Becton-Dickinson PrecisionGlide^® ^needles, 16 and 18 gauge (table 1), were prepared for punching the donor blocks, as follows: we used a Dremel Multi-Pro^® ^Rotary Tool Model 395, at low speed (< 10,000 rpm), with a cutting wheel attached, to cut off the bevel and straighten the tip. Using the same tool, lateral openings (windows) were made, 1 mm away from the new tip, with 8–10 mm length and slightly wider than half the needles' width, so that the punched core could be safely removed through it. Then the external surface of the needle was sharpened with the sanding surface of the cutting wheel. The internal surface was also sharpened using either a shaft cone stone or a metal sharpener. Cores were manually punched out from the donor blocks, from regions selected on correspondent Hematoxylin-Eosin (H&E) slides overlaid. At this step it is important that the needle enters the block in a perpendicular way, so that the cores extracted will have a perfectly cylindrical shape. We alternatively adapted a hand-press grommet inserting machine, by means of removing the dies, and attaching one of the customized needles in their place, which ensures an even easier semi-automated movement, as well as a perpendicular angle between the needle and the donor block. The total initial cost was near USD$100.00 (table 2).

**Table 1:** Needles measures and characteristics

**External diameter**		**Hub color**	**Internal diameter**		**Core area (mm^2^)**
			
**Gauge**	**Millimeter**		**Inches**	**Millimeter**	
16 G1 1/2	1,60 × 40	white	0.047	1,19	1,1
18 G1 1/2	1,20 × 40	pink	0.033	0,83	0,53
21 G1 1/4	0,80 × 30	green	0.020	0,5	0,2

**Table 2:** Costs

**Item**	**Unitary cost US$**
DREMEL Multi-Pro^® ^Rotary Tool Model 395	80.00
Hypodermic BD PrecisionGlide^® ^needles	1.00
Double-face adhesive tape, Scotch 3 M^®^	2.00
Hand-press grommet inserting machine	17.00
Total	100.00

### Paper grid (figure 1)

We designed a grid using the drawing software Corel Draw^® ^the following way: 1 mm white circles were drawn and aligned leaving 0,5 to 1 mm space between them, on a colorful background; the grid was printed in plain paper.

### Mould (figure 2)

**Figure 2:**
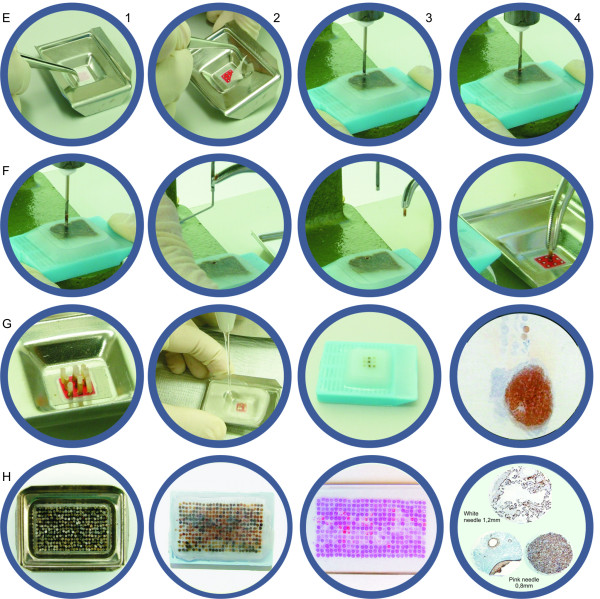
**TMA block production and examples**. TMA block production, step by step – grid positioning with double-side adhesive tape (E1 and E2); punching tissue core with hand-press grommet inserting machine (E3 to F3); positioning tissue core on the grid, over the double-side adhesive tape (F4); 3 × 3 grid complete with 9 tissue cores in the mould (G1); pouring liquid paraffin into the mould (G2); TMA block with 9 tissue cores (G3); slide with 9 tissue cores TMA for IHC control + case section [IHC with chromogranin A primary antibody, carcinoid tumor] (G4); 25 × 13 grid complete with 325 tissue cores in the mould (H1); TMA block with 325 tissue cores, 25 × 13 grid (H2); H&E slide with 325 tissue cores, 25 × 13 grid (H3); immunohistochemistry stains in cores done with 16G needle [PLAP, placenta, 4×] and 18G needle [cytokeratin AE1/AE3, teratoma, 4× and CD3, tonsil, 4×] (H4).

Paper grids were attached to stainless steel moulds by means of a double-face adhesive tape, Scotch 3 M^®^, 12 mm wide. The tape should be wider than the grid, so that the free border of the adhesive tape bottom surface could attach the paper grid into the mould and the whole upper surface should be available for attaching the tissue cores. Following, the extracted cores were attached to the tape, overlying the selected white circles on the paper grid, in an orderly fashion. We used 7,0 × 7,0 mm, 15,0 × 15,0 mm and 25,0 × 15,0 mm moulds, according to the number of cylinders desired.

### Blocks (figure 2)

Once all cores were attached to the mould, melted paraffin was gently poured into the mould. It is important to make sure that all cores are perfectly vertical at this step. From this point, blocks were handled according to routine histopathological procedures. Sections were cut on an American Optical standard rotatory microtome, 4 μm thick; each block provided 90–100 slides, depending on the tissue cores' length. There was no need to use adhesive-coated tape-sectioning. The design of each block was detailed in a TMA map, indicating the position and identification of each core. We used normal tissues, different sized cores or position-specific blank cores for orientation during microscopy.

### Slides (figure 2)

Hematoxylin-eosin (H&E) stained slides were obtained and processed in the conventional way. IHC was performed using standard avidin-biotin-peroxidase complex manual procedures using slides pre-treated with silane (3 amino-propyl-trietoxysilane) 3% in acetone, heat-mediated antigen retrieval by incubating slides in hot water bath at 96°C/204.8°F for 40 minutes and DAB as chromogen.

## Results

### Needles

Crafting the needles was easy and quick, requiring little skill to produce the bevel and lateral window – after a short period of learning and training (a few hours), everyone was able to produce good quality needles in less than 15 minutes. It is advisable to use protection eyeglasses and gloves in order to avoid harm from metal dust.

### TMA blocks

The construction of TMA blocks using this technique was also quick (30 minutes to make one 25-cores block) and easy (once the exact areas are marked on H&E slides, the technician can punch the donor blocks and construct the TMA block following the designed map). This approach is interesting because with only a few conventional histopathology paraffin donor blocks, several almost identical TMA blocks can be done, and hundreds or thousands of slides obtained.

### Immunohistochemistry

Initially, 79 different primary antibodies, monoclonal and polyclonal, were tested successfully for tittering/protocol establishment (75 from Biogenex^® ^and 4 from Novocastra^®^) using 320 slides from TMA blocks containing 25 different samples from normal or neoplastic tissues. After this antibody tittering/protocol establishment phase, several smaller TMA blocks (9 tissue cores, 3 × 3 grid) were designed to be used as routine IHC positive and negative controls, each one for a group of primary antibodies. One TMA block with 9 tissue cores (3 × 3 grid) was designed to be used as histochemistry/special stains positive and negative controls. Each block was cut until one tissue core finished, resulting in 90–100 control slides. These control slides were stored in plastic slide boxes at 4°C/39.2°F for no longer than 4 weeks. Routinely, each IHC case slide had two areas: the control TMA just below slide identification and the case section just below the control TMA (figure 2). More than 200 IHC control TMA blocks with 9 cores (3 × 3 grid) have been confectioned up to the present. Larger TMA blocks have been produced using this technique for research or demonstration purposes, ranging from 81 (9 × 9 grid) to 325 cores (25 × 13 grid, figure 2). There were little core losses (<1%), most of them due to consumption of one or more tissue cores during microtomy, rarely to section falling off from slides during technical procedures. To avoid this irregular consumption, it is important to punch cores with near the same tissue length, so one should avoid donor blocks from which many slides have been cut or that bear too thin tissue specimens (at least 2 mm thick). Both 16 and 18 gauge needles yielded the same satisfactory results.

## Discussion

Recently there have been reports of alternative methods for the manual construction of tissue microarrays [4-7]. This article describes another alternative that have been tested for over one year, which is inexpensive, easy to reproduce and quick to perform, and produces high-density aligned TMA blocks that can be manipulated the same way any other paraffin tissue block. Following the present method, blocks do not break apart and there is a minimum core loss, precluding the use of an adhesive tape sectioning device. The initial cost is near USD$100.00; additional costs are negligible and include solely the eventual purchase of extra hypodermic needles for customization and double-face adhesive tape. It is also possible to use bone marrow trephine biopsy needles or other types of needles, however hypodermic customized needles have proven to be much more cost-effective, and the lateral opening is useful. The use of the double-face adhesive tape to attach cores was recently described [8], and we have developed a grid that allows for a satisfactory alignment of tissue fragments, and blocks thus obtained necessarily display tissue cores aligned and at the same cutting plane. Although the needles can be used manually, using the hand-press grommet inserting machine makes core punching easier, once it is absolutely perpendicular, prevents damage to the donor blocks and is faster.

## Conclusion

We present an alternative method for the construction of high-density tissue microarray blocks that can be performed by any anatomic pathology laboratory, at low cost and requiring minimum skill and time.

## Competing interests

The author(s) declare that they have no competing interests.

## Authors' contributions

All authors contributed equally to this work, in all its steps. All authors read and approved the final manuscript.
